# DEMENTIA CAREGIVING STYLES: USING EXPERT FEEDBACK TO ADAPT THE PARENTING STYLES MODEL

**DOI:** 10.1093/geroni/igae098.3375

**Published:** 2024-12-31

**Authors:** Darby Simon, Emilee Ertle, Benjamin Mast

**Affiliations:** Massachusetts General Hospital, Boston, Massachusetts, United States; University of Louisville, Louisville, Kentucky, United States; University of Louisville, Louisville, Kentucky, United States

## Abstract

Identifying how family caregivers provide care to people living with dementia has important implications for the quality of life for both parties. This project adapted the well-researched parenting styles framework to create a new measure reflecting Dementia Caregiving Styles. Although there are important differences between people with dementia and children, there are similarities between parents and family caregivers in how they approach these caring relationships. This creates comparable experiences such as balancing autonomy versus safety, making decisions on behalf of their care-recipient, and changing family roles. The current framework measures caregivers’ levels of responsiveness, demandingness, and autonomy-granting during interactions with their care-recipients. This poster reviews the process of adapting the Parenting Styles Inventory-II into the proposed Dementia Caregiving Styles Inventory by collecting feedback from dementia caregiving experts on this topic. For each item, experts rated the relevance to dementia caregiving and provided qualitative feedback. Experts could also provide their overall thoughts about adapting the parenting styles framework for dementia caregivers. Lynn’s (1986) standards were used to establish each item’s content validity via expert agreement. Several themes emerged from the experts’ feedback. Most notably, the demandingness subscale’s items required major revisions to apply to dementia caregiving. Conversely, the subscales regarding responsiveness to the care-recipient’s needs and balancing autonomy versus safety were applicable to dementia caregivers. At the end of the revision process, the experts were overwhelmingly in support of the items suggesting that adapting parenting styles to dementia caregiving could provide a framework that helps family caregivers provide better dementia care.

